# On or off topic? Understanding the effects of issue-related political targeted ads

**DOI:** 10.1080/1369118X.2023.2265978

**Published:** 2023-10-12

**Authors:** Xiaotong Chu, Lukas Otto, Rens Vliegenthart, Sophie Lecheler, Claes de Vreese, Sanne Kruikemeier

**Affiliations:** a University of Amsterdam, Amsterdam, Netherlands; b Wageningen University & Research, Wageningen, Netherlands; c GESIS, Cologne, Germany; d University of Vienna, Wien, Austria

**Keywords:** Data-driven campaigning, issue congruency, vote choice, mobile experience sampling method, panel survey

## Abstract

Whilst data-driven strategies are allegedly prevalent in political campaigns, evidence regarding their actual effectiveness is scarce. This study investigates, from an individual perspective, the effect of issue congruency in political ads on immediate responses and voting behaviors. To reach our goal, we combined different types of data collection: mobile experience sampling method (mESM), panel survey, and content analysis. The combined approach allowed us to effectively study targeted ads within the cross-device and cross-platform environment. The results showed that voters perceive online political ads that are about a topic that they care about as more interesting, informative, and persuasive regardless of their partisanship. This positive ad perception subsequently leads to a higher probability of voting for the promoted party in the ad. We also found that an ad discussing a topic in line with the receiver’s concerns positively affects the evaluation of the promoted party in the ad only when the party is already favored by the voter. Taken together, this study provides insights into the conditional effectiveness of data-driven strategies in political campaigns.

Political competition is about campaign-voter alignment. Nowadays, by means of garnering and analyzing electorates’ personal data on social media, modern campaigns can identify groups of audiences and thus disseminate tailored ads to specific segments of the public (Dommett, 2019). This campaigning practice is labeled data driven. Previous studies have examined data-driven campaign messages tailored to different groups, based on partisanship, religion (Alvarez et al., 2010; Hersh & Schaffner, 2013), demographics (Haenschen & Jennings, 2019; Ostfeld, 2017), and psychometrics (Matz, 2021). Within data-driven campaigns, focusing on issue appeals is one of the most used strategies (Endres, 2020). The aim is to target voters with an issue congruent with their interests or concerns (Endres, 2020; Kim et al., 2018). For example, compared to younger citizens, senior citizens may receive more political ads regarding pension policies. Issue congruency indicates whether the advertised topic in a political campaign message matches an individual’s concerns about social and political issues.

Targeting voters with issues is allegedly one of the most effective ways of data-driven campaigning (Kim et al., 2018). However, empirical research regarding the effects of issue congruency has been largely focused on electoral outcomes and is rather scarce and sometimes conflicting. Previous research has found that targeting voters with their concerns elicits positive responses such as political participation (Matthes & Marquart, 2015) and vote choice (Endres, 2020; Lavigne, 2020). However, mistargeting voters with issues they have no concerns about mutes the positive effects of data-driven campaigning (Hersh & Schaffner, 2013), or even results in negative electoral consequences and ‘backlash effects’ (Motta & Fowler, 2016). Previous work has mainly focused on the US two-party context, while limited attention has been paid to multiparty democracies. In the latter electoral context where political parties vary in visibility and political stances (Gibson & McAllister, 2015), the impact of issue-related campaigning may be different. In this study, we pose the question: in a multiparty system, how does issue congruency in political campaign messages affect vote choice? To answer this question, we seek to construct the understudied processes activated prior to voters’ actual voting behavior, such as voters’ perception of the ad and evaluation of the party (Herstein, 1981). In the meantime, voters’ preexisting attachment to the advertised party in political ads could possibly affect their cognitive processes and vote outcomes when confronted with the ads. Hence, we also investigate whether and how party identification plays a role in this process. In sum, this study attempts to coherently examine voters’ immediate responses (i.e., ad perception, and party evaluation) and behavioral responses (i.e., vote choice) toward issue congruency in political advertising messages.

Mobile devices are an ideal venue to study data-driven campaigning and targeting strategies. First, most (personalized) social media communications nowadays are largely mobile (Kuru et al., 2017). Second, smartphones provide campaigners with voters’ mobile digital data such as browsing behaviors, locations, mobility, and other sensor data (Brandtzaeg et al., 2019). Third, mobile phones can come in handy as online users can easily take a screenshot or a photo of any online information they encounter. Therefore, to answer our research question, a mobile Intensive Longitudinal Linkage Analysis (MILLA) approach (Otto et al., 2022) was conducted in the lead-up to the 2021 Dutch General Election.

## Issue-related targeting strategies in data-driven campaigning

Although targeting voters with personalized information is hardly a new campaign tactic, this strategy has been reckoned to be more recurrent and personalized in recent years due to the use of technology in political campaigns (Dommett, 2019; Ryabtsev, 2020). The core of data-driven campaigning lies in the party-voter alignment, meaning that voters receive campaign messages with appeals that are supposed to match their partisanship, personal characteristics, or interests. As the competition among political parties has progressively evolved into issue competition, targeting voters with their interests or concerns has become the most effective approach to mobilize and persuade voters (Van der Brug, 2004).

During the past decades, the explanatory power of issue-related theories on campaigns and voting behavior has broadly speaking shifted across three phases: issue position, issue salience, and issue yield. The effect of a party’s issue positions in elections was first proposed by Downs (1957). His proximity model assumed voters simply vote for the party that falls the closest to their own positions. Such assertion was challenged by other scholars because confrontations between policy positions are quite rare in real-life elections (Stokes, 1963). They argue that voters normally do not have a clear picture of policy positions but rather a general desire regarding a certain issue and a direction, for instance, a cleaner environment (Rabinowitz & Macdonald, 1989). In response to the critiques toward issue position being the greatest predictor of electoral success, issue salience is the central element in the prominent issue ownership theory (Budge & Farlie, 1983; Petrocik, 1996) and relates strongly to the classical notion of agenda setting. Issue salience, also known as issue priority and issue emphasis, refers to a campaign impact that occurs when a party selectively emphasizes a small set of issues that they have a greater commitment to resolve. By prioritizing favorable issues and downplaying unfavorable issues, political parties link themselves to specific issues and claim ownership (Tresch et al., 2018). However, this theory failed to take voters’ perceptions of issues into consideration (Van der Brug, 2004). Recently, De Sio (2017) has proposed the issue yield theory, which has been tested in multiparty systems (De Sio & Weber, 2020). The theory posits that an election outcome can be predicted by summarizing both the electoral opportunities and risks of issues while considering the overall electorates’ preference. However, as issue yield theory evaluates issue-related campaigns from a party perspective, the reciprocal dynamics of communications between the party and voters are neglected. Moreover, as voters’ issue preferences are assessed jointly, the nuances between individual voters are not considered.

Literature nowadays largely focuses on issue positions. This study, however, adopts a salience-based approach. We consider congruency, in terms of salience, a valid mechanism to account for differential impacts of campaign messages, as we will explicate below. We define issue congruency as whether an issue described in a campaign message matches the democratic issues that are concerns of the message recipients. As issue targeting is individual-level campaigning, we attempt to examine issue congruency between individual voters and the political ad they receive. In other words, instead of seeing issue congruency as an overall perception of targeting at a campaign level, this study reckons issue congruency as a dynamic phenomenon that can be established individually when a voter is exposed to a political ad.

## The persuasiveness of issue congruency

Issues as the core of political products have played an important role in predicting the election outcome. Issue voting refers to the extent to which voters cast their ballot based on political issues, which involves voter mobilization and persuasion (Endres, 2020; Van der Brug, 2004). Mobilization has been excessively studied in strategic campaigning research (Burge et al., 2020; Cann & Cole, 2011; Haenschen, 2022). However, due to the complex nature of political persuasion, the persuasiveness of political campaigning needs more attention, especially in multiparty contexts. In terms of the effect of strategic campaigning in general, scholars found that political campaigns have a very small persuasion effect. Kalla and Broockman (2018) conducted a systematic meta-analysis of 49 field experiments, and they found an average effect of zero in general elections. Other studies also confirmed the small effects of campaigning (Coppock et al., 2020; Coppock et al., 2022).

In terms of targeting campaigning, the utilization of a data-driven strategy in election campaigns may help to maximize votes effectively (Kenski et al., 2010). Although Kalla and Broockman (2018) found almost zero average persuasiveness in political campaigns, they also found positive effects when targeting is present. With a special focus on issue congruency, the self-congruity theory (Sirgy, 1986) originating in the field of marketing communication provides support for the framework where the fit between an individual and promoted product positively impacts the behavioral outcome. This behavioral effect of targeting strategy has been supported by Schmidt and Hitchon (1999), and they found that, compared to ads mentioning an incongruent issue, ads discussing a congruent issue tend to provoke favorable responses and increase the likelihood to purchase the advertised product. Other marketing studies also supported the assumption that ads accurately targeted individuals’ interests and increase click-through rates (Aguirre et al., 2015) and purchase behavior (for an overview, see Boerman et al., 2017). In the political context, evidence supports that a congruent message has a greater impact than a non-congruent message. Matthes and Marquart (2015) found that like-minded political advertising can improve political participation. Van der Brug (2004) also proved the persuasiveness of issue targeting, even though to a very limited extent. Therefore, we expect a positive effect of issue congruency on vote choice.

H1: Exposure to a political ad with a congruent issue, compared to a political ad with an incongruent issue, leads to a higher likelihood to vote for the promoted party in the ad.

## Effect of issue congruency on ad perception and party evaluation

Previous research is rather skeptical about the impact of ads on election outcomes. Kalla and Broockman (2018), for example, pointed out that the impact of strategic communications on election outcome is very small, and sometimes not observed, because voters are overloaded with all kinds of campaign frames and arguments before the election day arrives, and thus a single-time exposure to a political campaign message is hardly effective. Campaign outcomes should not be simplified to merely election results. As the impact on the long-term voting decision may decay, it is worthwhile to study the immediate effect of political campaigning, as it could help us to understand the cumulative effects of political campaigning strategies. Although studying such immediate responses allows us to disentangle the impact of data-driven campaigning on election outcomes, we still lack empirical evidence of the immediate impacts of targeting messages. For this paper, we focus on the immediate responses of voters in two aspects: cognitive perception of political ads, and affective evaluation of the advertised party in the ads (MacKenzle et al., 1986).

We define ad perception as a perceptual judgment of ad characteristics (Daignault et al., 2013). The Elaboration Likelihood Model (ELM) (Petty et al., 1983) highlighted the important role of individual involvement, which is conceptualized as the extent to which an individual perceives the advertised object as relevant to what they care for. Petty et al. (1983) proposed that an ad congruent with an individual’s personal interest leads to a more favorable cognitive response toward the ad. Similarly, Lang (2006) found that congruent messages help to generate cognitive involvement, which subsequently facilitates how the message is processed. Voters in multiparty systems often encounter a variety of information from different sources before elections, their limited capacity for processing information only allows them to interact with messages highly involved or relevant to themselves. Therefore, messages with a topic congruent to their concerns or interests are perceived as more interesting and informative.

To translate cognitive effects to behavioral outcomes, Wright (1973) first proposed the mediating role of cognition in the process of advertising. Similarly, MacKenzle et al. (1986) proposed a dual mediation model, and they found that a persuasive message affects behavioral outcomes through the mediating effect of cognitive responses. The work by Bleier and Eisenbeiss (2015) provided evidence that personalized ads can increase people’s perceived usefulness of the ads, and this effect may directly translate into purchase behavior. Lang (2006) offered another explanation for this mediating effect. She argued that congruent messages tend to interest people, which subsequently leads to better information storage and retrieval at a decision point. As a consequence, we expect that voters perceive more relevant ads as useful and interesting, and thus such information is more likely to ring a bell when voters cast their vote. The following hypotheses are derived.

H2a: A political ad with a congruent issue, compared to a political ad with an incongruent issue, is perceived more positively.

H2b: Issue congruency positively affects ad perception, which in its turn positively affects vote choice.

Lutz and MacKenzle (1983) developed a model and distinguished between underlying responses toward ads and toward advertised objects. The model proposed that advertising has an impact on purchase behavior via ad perception and brand evaluation. Party evaluation is an effective response that indicates voters’ feelings toward a party (Rosenberg et al., 1960). Erdogan (1999) found that when exposed to a persuasive message, people view the advertised object that has a resemblance to themselves as more likable and trustworthy. In the political realm, an online experiment conducted by Binder et al. (2022) proved that political ads with a better fit lead to a more positive party evaluation. Likewise, Krotzek (2019) conducted a U.S.-based online experiment and demonstrated that congruent ads indeed lead to a more positive feeling toward the candidate. This study did not find evidence proving that this effect can translate to voting behavior, but a study that analyzed data from a Dutch election showed that favorable evaluations of the candidate and party yield higher vote intention (Takens et al., 2015). Given our specific interest in multiparty contexts, we expect the following.

H3a: Exposure to a political ad with a congruent issue, compared to a political ad with an incongruent issue, leads to a more positive evaluation of the promoted party in the ad.

H3b: Issue congruency positively affects party evaluation, which in its turn positively affects vote choice.

## The moderating role of party identification

Party identification might play a crucial role in explaining the effect of targeting. Party identification is defined as a sense of ‘attachment to a political party’ (Goren, 2005, p. 881), which is an inevitable factor when studying political persuasion. It is necessary to distinguish the two concepts: party evaluation and party identification. Party evaluation as an effective response reflects voters’ feelings toward parties, while party identification is linked to group belonging and social identity (Rosema, 2006). We argue that party identification as the premise of self-concept sets the tone for the direction and the extent to which issue congruency affects ad perception. On the one hand, a political ad promoting a self-identified party is more likely to advocate a policy position in line with the voter. According to the proximity theory (Downs, 1957), a party’s policy position can alter voters’ assessment. The immediate effects of a campaign message can be affected by not only the presence of a congruent issue but also the extent to which a policy position embedded in the message shares common ground with the recipients. When both the issue salience and position align with an individual’s concerns and opinions, a more positive perception of the ad, as well as a better evaluation of the party, can be expected (Matthes & Marquart, 2015). On the other hand, partisanship has a positive impact on responses to persuasive cues as self-identified parties tend to be seen as more trustworthy (Hooghe, 2020). Targeting ads often elicits people’s perceived privacy invasiveness, but ads promoting a trustworthy object can alleviate this negative feeling (Bleier & Eisenbeiss, 2015). Therefore, ads promoting a favorable party lead to a more positive ad perception and party evaluation, while ads targeting supporters of the opposing parties can result in penalization (Hersh & Schaffner, 2013). We hypothesize the following.

H2c: Party identification positively moderates the relationship between issue congruency and ad perception. Specifically, a congruent ad leads to a more positive ad perception when the ad promotes a more favorable rated party.

H3c: Party identification positively moderates the relationship between issue congruency and party evaluation. Specifically, a congruent ad leads to a more positive party evaluation when the ad promotes a more favorable rated party.

**Figure 1.**
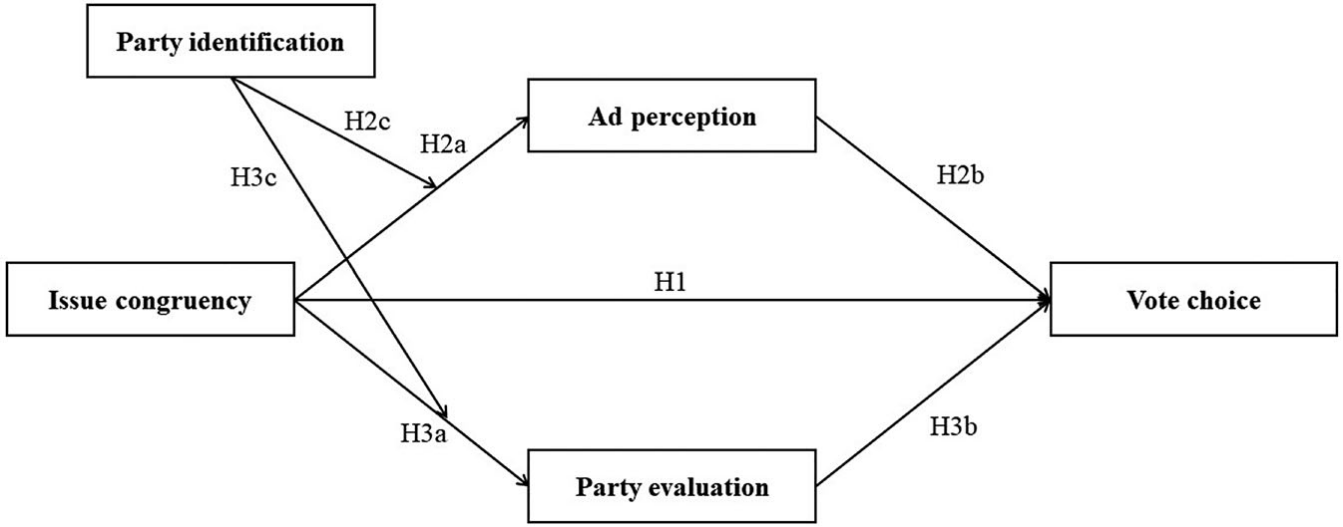
Conceptual model.

In sum, we assume a positive effect of issue congruency on immediate responses toward the ad and promoted party, which subsequently has a positive effect on voting behavior. Figure 1 shows the conceptual model.

## Method

**Figure 2.**
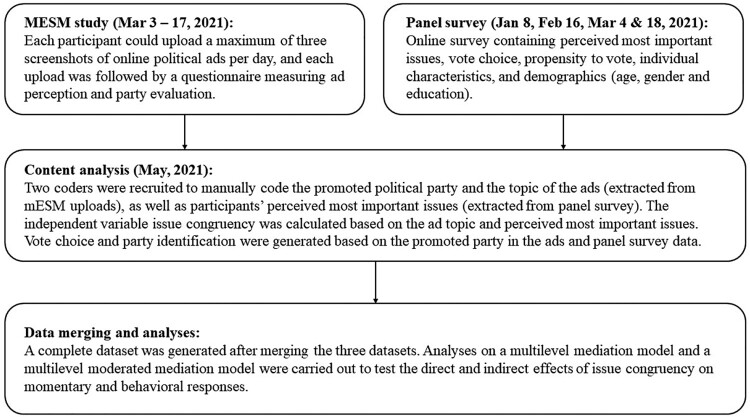
Flow diagram of the procedure.

To test our hypotheses, we used an approach that allows us to record accurate media use and issue congruency as well as capture immediate responses and vote choice. We adopted the following approach to testing our hypotheses. First, a panel survey was used to measure voters’ characteristics and their voting choice in the election, which was conducted throughout a period of two months before the 2021 Dutch General Election (see Figure 2). Second, a mobile experience sampling method (mESM) with an event-contingent sampling design was carried out throughout the two weeks in the run up to the election day. The mESM allowed us to capture voters’ online advertising exposure and their immediate responses from an individual-based perspective within a cross-device (i.e., mobile phone, tablet, and desktop) and cross-platform (i.e., Facebook, Instagram, news websites, etc.) environment. Third, a content analysis was conducted to code the ad sources and issues embedded in the ads. The Netherlands applies a multiparty and proportional electoral system. In 2021, 37 parties participated in the General Election, and 17 parties were elected to compose the new parliament.

### Participants

For both the mESM and panel survey study, data collection was approved by the Ethics Review Board of The University of Amsterdam (project filed as 2021-PCJ-13104). Participants were recruited via an audience research organization I&O for the panel survey. Out of the survey pool, 155 participants also signed up to take part in the mESM study. In the end, 140 participants uploaded online political ads and finished the panel survey. They were properly compensated for both the survey and mESM studies. 48.6% were female, and 51.4% were male. The average age of participants was 47.64 (SD = 16.27), and 72.1 percent held a bachelor’s degree or higher.

### mESM: political ads and immediate responses

An mESM study took place from 3–17 March to 2021. The last two weeks before the election day is often regarded as the most crucial phase of an election campaign (Ridout et al., 2021). The mESM provided us with an opportunity to capture participants’ actual and daily exposures to online political ads, and thus reduced memory bias caused by self-reported measures of ad exposures in the post-election phase. Besides, this method allowed us to study underlying psychological mechanisms (i.e., cognition) following contingencies in real-life settings.

Before the start of the study, participants were informed about the procedure of the study and gave consent to participate in the study. We provided participants with detailed instructions and had them thoroughly trained regarding the procedure of the study. We asked participants to take either a screenshot or a photo of online political ads they encountered on any device, and then upload them via a link sent to their phones every morning (see *Appendix E*). The maximum number of uploads was set to three per day to avoid overburdening the participants. After each upload, participants filled in a short questionnaire regarding their immediate responses to the ad (i.e., ad perceptions, ad-evoked emotions, and party evaluation). To nudge participants to continue participating, reminders were sent out every morning. In the end, 2,498 screenshots/pictures were uploaded. On average, each participant uploaded 1.07 ads per day (*SD *= 1.27).

### Panel survey: issues, individual characteristics, and voting behavior

A panel survey was designed to measure what voters perceived to be the most important issues in society, background characteristics, party identification, and vote choice. Surveys were conducted in four waves, respectively on 18 January, 16 February, 4 March (before the Dutch Election, and one day after the start of mESM study), and 18 March (in the aftermath of the Dutch Election, and one day after the end of mESM study). In every wave, participants were asked to list the most important issues facing the Netherlands, as well as indicate their vote choice, party identification, and demographic information.

### Content analysis: issue identification

To extract issue congruency between participants’ interest and the actual content of the uploaded online political ads, a content analysis was employed in May 2021. Two native Dutch bachelor students worked as the coders, and manually coded two datasets: the characteristics of both the texts and images in the uploaded screenshots from the mESM study (i.e., whether the ad was qualified as an online political ad, platform, publisher, promoted parties in the ad, and ad topic) and participants’ answers to the open question regarding most important issues. The unit of analysis of the former dataset was the uploaded screenshot, while that of the latter dataset was the participant. The coding started after coders familiarized themselves with the codebook and discussed the potential concerns regarding the coding procedure. A test of inter-coder reliability was performed for both datasets. 10 percent of both datasets were randomly chosen and coded by the coders. The average inter-coder reliability among all the issues was satisfying for both the mESM data (*Krippendorff’s α *= .75) and the panel survey data (*Krippendorff’s α *= .88) (Krippendorff, 2004).

After the pilot coding, two coders were assigned to code the rest of the datasets. The first step in coding the mESM data was to identify whether the uploaded screenshots were indeed online political ads. According to the coding, among the 2,498 screenshots uploaded by the participants, 2,035 were online political ads. The promoted party(ies) in the ad were also coded. 2,025 ads promoted one party, and 10 ads promoted more than one party. Afterward, the topics of political ads were coded. In the original codebook, we categorized issues into sixteen categories (Government of the Netherlands, 2021; Stier et al., 2018). Based on the content analysis, eight other issue categories were added to the list of issues (see *Appendix B*). It should be noted that an ad could consist of either no concrete topic or one or multiple topics. After coding, 1,017 ads contained no specific topic, 922 ads contained one topic, and 96 ads contained more than one topic. Likewise, the most important issues participants cared about were also coded into the 24 categories.

### Measures

#### Issue congruency

In all four waves of the panel survey, participants were asked to give answers to an open question ‘What do you think are the most important problems facing our country right now?’ (see *Appendix A*). Participants were allowed to name a list of issues that were later manually coded into 24 different categories (see *Appendix B*). We then combined all the coded issues of all four waves and generated a list of unique issues for each participant. For each uploaded online political ad, we calculated a congruency score (*M *= 0.22, *SD *= 0.41) based on the topic of the ad and the list of issues. Specifically, if the topic of the political ad appears in the list of issues, the ad was considered congruent and scored one on issue congruency; if the topic aligned with none of the issues the participant was concerned about, the ad was reckoned as incongruent and thus scored zero on issue congruency. In the end, 446 ads contained a topic congruent with the participant’s perceived most important issue.

#### Ad perceptions

Ad perceptions were measured by asking the participants to what extent they find the uploaded ad interesting, informative, and persuasive on a 7-point Likert scale ranging from 1 (not at all) to 7 (very much) (Sundar & Kim, 2005). Ad perceptions were calculated by averaging the scores of the three items (*Cronbach’s α *= .95, *M *= 3.05, *SD *= 1.60).

#### Party evaluations

To measure party evaluations, participants were asked to indicate to what extent they find the party in the uploaded ad honest, competent, and likable on a 7-point Likert scale ranging from 1 (not at all) to 7 (very much). Party evaluations were calculated as an average of the three items (*Cronbach’s α *= .94, *M *= 3.58, *SD *= 1.53).

#### Vote choice

Vote choice (*M *= 0.13, *SD *= 0.34) describes the actual vote choice in the election. Participants were asked to select one party from the list of eighteen Dutch political parties. This list consists of thirteen established parties that won seats in the 2017 Dutch General Election, and five newcomers founded after the 2017 election (see *Appendix D*). Participants could also name a party if the party they intended to vote for was not listed. A dichotomous variable was constructed: if the final vote choice matched the promoted party in the ad, the score was assigned as 1; if the final vote choice did not match the promoted party in the ad, the score was 0.

#### Party identification

Propensity-to-vote was used as a proxy of party identification (*M *= 3.96, *SD *= 3.62), which describes the likelihood to vote for a party and is relatively stable over time (Van der Eijk & Niemoller, 1984). In the third wave of the panel survey (the closest wave to the start of the mESM study), participants were asked to indicate how likely they will vote for a political party in the General Election on a 12-point scale (0 = I will never vote for this party, 11 = I will definitely vote for this party). They needed to answer this question for each one of the eighteen Dutch political parties. We then used the propensity-to-vote score of the promoted party in the ad as party identification. If more than one party were promoted in an ad, the average score of propensity-to-vote would be calculated.

#### Control variables

People among different demographic groups may have their expertise in different issue domains. As our sample was not representative, we controlled for participants’ demographic information (i.e., age, gender, and education level) (see *Appendix A*).

### Descriptive results on issue-related targeting practices

**Figure 3.**
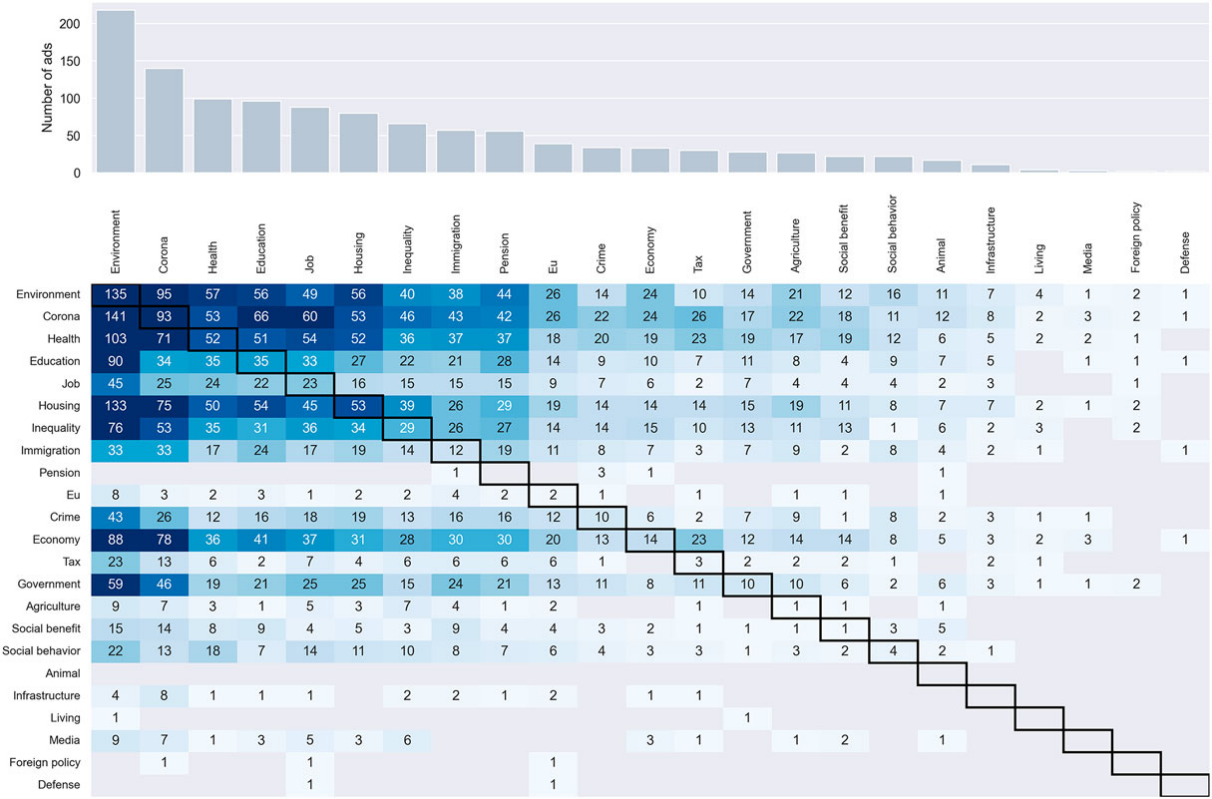
The matrix of the number of topics in political ads and participants perceived most important issues. Note. The X-axis indicates the topics, Y-axis indicates participants perceived most important issues.

Regarding the salience of topics, Figure 3 shows a heatmap of topics in political ads across participants perceived most important issues. Specifically, environment and COVID-19-related issues were the two most mentioned topics in online political ads. It should be noticed that the 2021 Dutch Election happened in the universal wave of ‘climate elections’ Wilson (2022) and that this narrative was pronounced by many political elites during the election campaigns (Vrancken, 2022). Also, this election particularly happened in the midst of the COVID-19 pandemic. This explains why these two topics not only appeared significantly more than other topics in the campaigns but also the biggest concerns of most of the participants. Furthermore, we observed that participants who perceived a certain issue as more important did not necessarily receive more online political ads regarding that issue. We also found that participants received ads from diverse parties (see *Appendix I*). In other words, we did not observe evidence of political parties targeting voters during the 2021 Dutch Election campaigns. Other detailed descriptive information regarding the data donation can be found in *Appendix C and Appendix I*.

## Hypotheses testing

### Analytic strategies

As repeated measures were taken per participant, our data had a hierarchical structure. We calculated the intra-class correlation (ICC) with null models for all the variables. The results showed that ICC for the congruency level, ad perception, party evaluation, vote choice, and party identification were respectively .07, .39, .34, .13, and .24, which indicated that a relatively high variation in our outcome variables could be explained by differences between individual characteristics. Therefore, we carried out two multilevel path analyses using the R package Lavaan (version 0.6-11; Rosseel, 2012). Bivariate correlations between independent and dependent variables were also reported in *Appendix F*.

A multilevel mediation model (*X*^2^(3) = 2.735, *p *= .434; *CFI *= 1.000; *RMSEA *= .000; *SRMR *= .000) was conducted to test the direct effects of issue congruency, as well as the mediating effects of ad perception and party evaluation on vote choice. A multilevel moderated mediation model (*X*^2^(5) = 511.583, *p *< .001; *CFI *= .858; *RMSEA *= .170; *SRMR *= .065) was carried out to test the moderating role of party identification on the effect of issue congruency. All the parameters were estimated with the standard 1-1-1 mediation model, meaning that all the central variables in the conceptual model were measured or calculated based on each ad exposure, and thus they were at the within-individual level (level 1). These central variables included the predictor issue congruency, the mediators ad perception, and party evaluation, the outcome variable vote choice, and the moderator party identification. Our control variables, however, were all measured at the between-individual level (level 2), including demographic information age, gender, and education. The Lavaan syntax can be found in *Appendix G*.

All parameter estimates of the multilevel mediation and multilevel moderated mediation model are summarized in Table 1. At the within-individual level, we compared the effects of each ad exposure on a single response of an individual. At the between-individual level, we compared the systematic differences among individuals’ general responses to a set of ads they were exposed to. Please note that as our hypotheses focus on issue congruency and evoked responses of every single exposure to one online political ad, we only interpreted the results at the within-individual level. Five independent multilevel regression analyses were also conducted using STATA as a robustness check, and similar findings were reported (see *Appendix H*).

**Table 1.** Multilevel models predicting vote choice with ad perception and party evaluation as mediators.

Variables	Multilevel mediation model		Multilevel moderated mediation model	
	B	SE	B	SE
*Within-individual level*				
Outcome: Ad perception				
Congruency	0.260***	0.072	0.136	0.092
Party identification			0.195***	0.009
Congruency * Party identification			0.028	0.017
Outcome: Party evaluation				
Congruency	−0.039	0.073	−0.262***	0.078
Party identification			0.271***	0.007
Congruency * Party identification			0.053***	0.014
Outcome: Vote choice				
Congruency	0.004	0.017	0.005	0.018
Party identification	0.029***	0.008	0.030***	0.008
Congruency * Party identification	0.080***	0.007	0.082***	0.008
				
*Between-individual level*				
Outcome: Ad perception				
Congruency	−0.879	1.216	0.164	1.793
Party identification			0.229**	0.084
Congruency * Party identification			0.097	0.351
Age	0.002	0.007	0.008	0.006
Gender	−0.172	0.202	−0.084	0.194
Education	−0.366	0.181	−0.317	0.174
Outcome: Party evaluation				
Congruency	−1.015	1.116	−0.525	1.598
Party identification			0.248***	0.073
Congruency * Party identification			0.089	0.311
Age	−0.005	0.006	0.001	0.005
Gender	−0.312	0.183	−0.225	0.164
Education	−0.077	0.164	−0.026	0.147
Outcome: Vote choice				
Congruency	−0.237	0.161	−0.337	0.240
Party identification	−0.034	0.023	−0.028	0.024
Congruency * Party identification	0.086**	0.027	0.075**	0.024
Age	0.001	0.001	0.001	0.001
Gender	−0.028	0.027	−0.029	0.027
Education	0.006	0.024	0.005	0.024
N of observations	2033		1935	
N of respondents	140		138	
Log likelihood user model	−7728.547		−15314.222	
Log likelihood unrestricted model	−7727.179		−15058.430	
Akaike (AIC)	15521.094		30708.444	
Bayesian (BIC)	15700.846		30931.158	

^∗^ *p < *.05; ^∗∗^*p < *.01; ^∗∗∗^*p < *.001.

### The effect of issue congruency on vote choice

First, we tested the direct effect of issue congruency on vote choice. As shown in Table 1, we found no direct effect of issue congruency on voters’ final vote choice at the within-individual level (*b *= 0.004, *SE *= 0.017, *p *= .823). The direct effect remained insignificant when we excluded the mediators from the model (see *Appendix H*). This showed that whether or not advertising an issue congruent with an individual’s concerns or interests (basically a ‘successfully’ targeted ad) did not affect vote choice. Therefore, H1 was not supported.

### The effect of issue congruency on ad perception and party evaluation

Second, we looked at the direct effect of issue congruency on individuals’ perception of online political ads. As shown in Table 1, the results demonstrated that issue congruency has a significantly positive effect on ad perception at the within-individual level (*b *= 0.260, *SE *= 0.072, *p *< .001). The effect of issue congruency on ad perception is relatively small, in the sense that a congruent ad can boost people’s ad perception by 4.33 percent, thereby supporting H2a.

We also tested the direct effect of issue congruency on the evaluation of the promoted party in online political ads. We found that the direct effect of issue congruency on party evaluation is not significant at the within-individual level (*b *= −0.039, *SE *= 0.073, *p *= .597), which means that an ad with a congruent topic does not lead to a more positive party evaluation. Therefore, H3a was not supported.

### The moderating effect of party identification

Third, we examined the moderating role of party identification. The results revealed no significant interacting effect for issue congruency and party identification on ad perception at the within-individual level (*b *= 0.028, *SE *= 0.017, *p *= .103). Therefore, whether or not an online political ad promotes a favorable party has no effect on the relationship between issue congruency and the way an individual perceives the ad. H2c was not supported.

**Figure 4.**
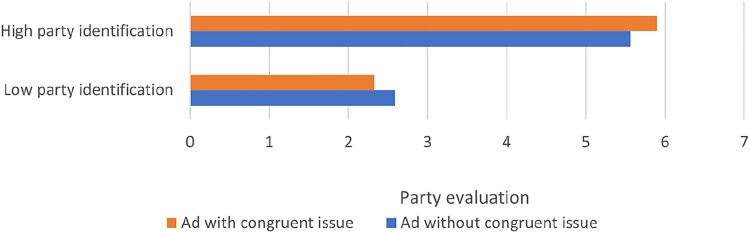
Moderating effect of party identification on the relationship between issue congruency and party evaluation (low party identification = 0, high party identification = 11).

The results showed a significant interacting effect of party identification on the relationship between issue congruency and party evaluation at the within-individual level (*b *= 0.053, *SE *= 0.014, *p *< .001). As shown in Figure 4, party identification moderates the effect of issue congruency on party evaluation, in the sense that a congruent ad leads to a more positive party evaluation only when the ad promotes a more favorable party, supporting H3c.

### The mediating effect of ad perception and party evaluation on vote choice

In addition, mediation analyses were conducted to test the indirect effect of issue congruency on vote choice via ad perception and party evaluation. At the within-individual level, the direct effect of issue congruency on vote choice (*b *= 0.004, *SE *= 0.017, *p *= .823, 95% CI [−0.029, 0.037]) and the total effect (*b *= 0.011, *SE *= 0.017, *p *= .504, 95% CI [−0.022, 0.044]) were both non-significant, while the indirect effect of issue congruency on vote choice via ad perception was significant (*b *= 0.007, *SE *= 0.003, *p *= .009, 95% CI [0.002, 0.013]). This demonstrates that ad perception mediated the relationship between issue congruency and vote choice, meaning that issue congruency positively affected ad perception, which subsequently had a positive influence on vote choice. H2b was supported.

We then tested the indirect effect of issue congruency on vote choice via party evaluation. The results showed that, at the within-individual level, the indirect effect of party evaluation was significant only when the ad promoted a favorable party (*b *= −0.021, *SE *= 0.007, *p *= .001, 95% CI [ −0.035, −0.008]), but both the direct effect of issue congruency on vote choice (*b *= 0.005, *SE *= 0.018, *p *= 0.760, 95% CI [−0.029, 0.040]) and the total effect (*b *= −0.016, *SE *= 0.019, *p *= .386, 95% CI [−0.053, 0.020]) was non-significant. This indicates that party evaluation helped to explain the process by which issue congruency influenced vote choice when considering the effect of party identification. In other words, when the ad promoted a favorable party, issue congruency had a positive effect on party evaluation, which subsequently affected vote choice. Therefore, H3b was partially supported.

## Discussion

This study investigates how issue congruency affects voters’ immediate responses and vote choice. We found that issue congruency has a positive effect on ad perception, meaning that voters found an online political ad with a topic related to their interest (a ‘successfully’ targeted ad based on issues) more interesting, informative, and persuasive. This positive effect of issue congruency on voters’ ad perception is not affected by political identification. In other words, an online political ad that is congruent on the basis of issues leads to positive feelings towards the ad regardless of partisanship. In addition, we also found that a congruent ad can only lead to a more positive party evaluation when the ad is disseminated by a more favorable party, while a congruent ad promoting a non-favorable party lowers the voter’s evaluation of the party. The results also demonstrated that ad perception and party evaluation play a mediating role in the relationship between issue congruency and vote choice. This indicates that vote choice can be predicted by a voter’s ad perception and party evaluation, and these immediate responses depend on whether the ad contains a topic that concerns the voter.

### Theoretical implications

By investigating the cognitive and affective responses toward political ads and parties, we provided more nuanced insights into the effects of political campaigns. The results confirmed the immediate effect of persuasive cues advised by the ELM, and the moderating role of identification suggested by the proximity theory. Specifically, an issue in line with personal interest indicates a higher involvement, which evokes a positive perception of the ad. A self-aligned party in the ad also generates a positive evaluation and alleviates a feeling of intrusion by online ads. This highlights the importance of studying the underlying mechanisms when building media effect theories.

Furthermore, this study opened the black box of data-driven campaigning. We found that voters were not massively exposed to online political ads in line with their concerns and interests, which confirms the arguments of previous research (Baldwin-Philippi, 2017; Endres, 2016). Our findings regarding the impacts of issue congruency also lent some indirect credence to the effects of data-driven campaigning with other targeted information, given that voters’ characteristics, such as demographics, partisanship, and psychometrics, are often related to their issue preferences. This study provided comprehensive insights into the immediate responses toward targeted messages, and how this effect translates to the persuasiveness of data-driven campaigning.

### Methodological implications

MILLA is a unique and innovative methodological advancement for researchers in the field. This study was the first attempt that applied this approach to a meaningful context, which indicated the feasibility of the combination of an experience sampling method with smartphone data donations following a questionnaire, a panel survey, and content analysis. This approach was introduced in response to the recognition of two obstacles in studying social media effects. First, advertisements on social media nowadays are tailored, fragmented, and hybrid. This determines the difficulty of accurately reporting news usage across different platforms and defining the level of personalization. Hence, surveys with self-reports conducted at the end of the election cycle could possibly raise memory errors, while experiments may result in simplifying the complex feature of online advertising. An experience sampling method with content analysis is particularly useful in this situation as it allowed us to access ads in the hybrid media landscape, and to offer a voter-based perspective of online ads. Second, it is challenging to record both people's immediate responses toward each ad exposure and their final voting decision in a real-life and real-time setting. MILLA provided the possibility of combining an experience sampling method and surveys. Future studies could also apply this approach in different circumstances.

### Limitations and future research

This study focused on comparing the differences in the effects of issue congruency and non-congruency, while future studies could study the extent to which ad topics are congruent to voters’ preferences. The MILLA method also has a few limitations. On one hand, the intensive nature of the mESM might limit the selection of participants, resulting in a relatively smaller sample size and bigger proportion of individuals with higher level of political interest. On the other hand, the repeated measurement strategy applied could possibly make participants pay unusual attention to ad exposure in their daily lives. Extra attention to ad exposure has an impact on how people process the information, as well as their final behavior (Yang & Smith, 2009). Future studies can apply other data donation methods, such as browser tracking, to reevaluate the effect of targeted ads. In addition, the persuasiveness of political rhetoric varies across different issues (Blumenau & Lauderdale, 2022). In a real-life setting, it is hardly possible to keep the rhetoric framing consistent or randomize the presence of issue congruency. Future studies can use experiments to control for the rhetoric feature across different issues.

## Figures and Tables

**Figure B1.**
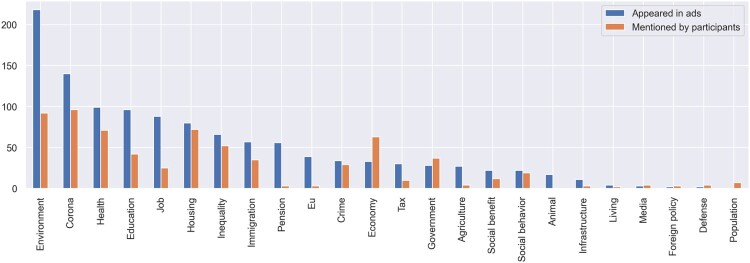
Number of issues appeared in Facebook political ads and mentioned by participants.

**Figure C1.**
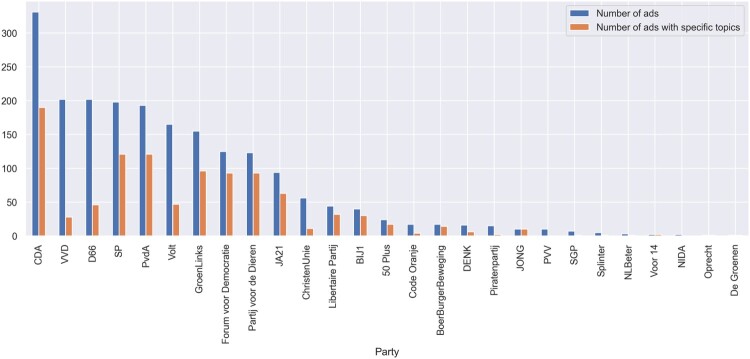
Number of ads and ads with specific topics per party.

**Figure D1.**
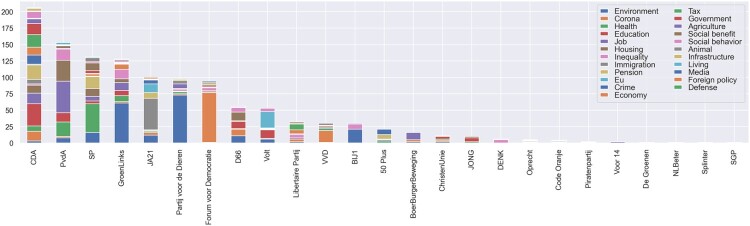
Topic salience per political party.

**Figure E1.**
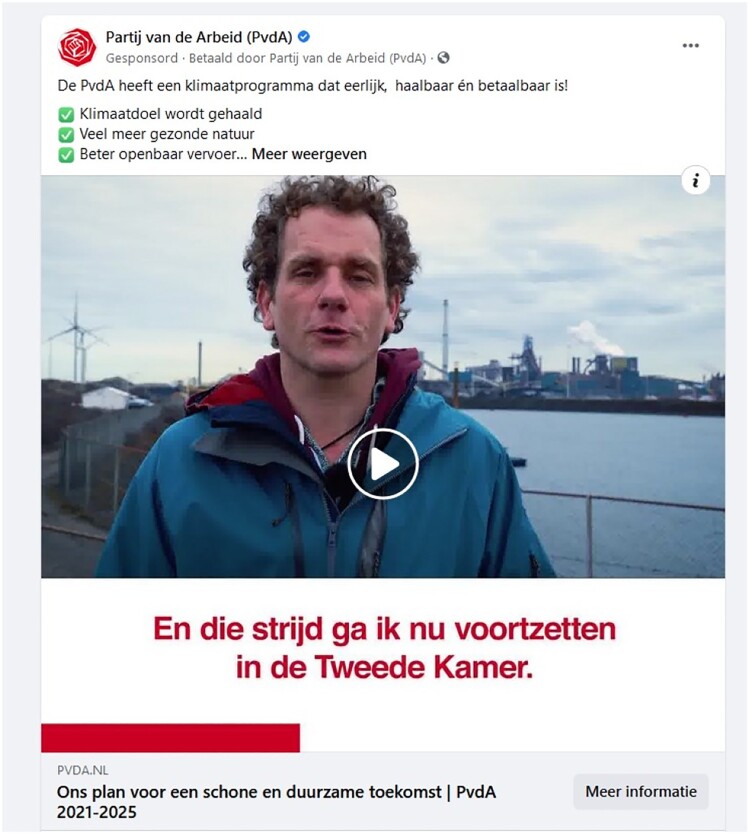
An example of an uploaded screenshot of a political ad.

**Figure I1.**
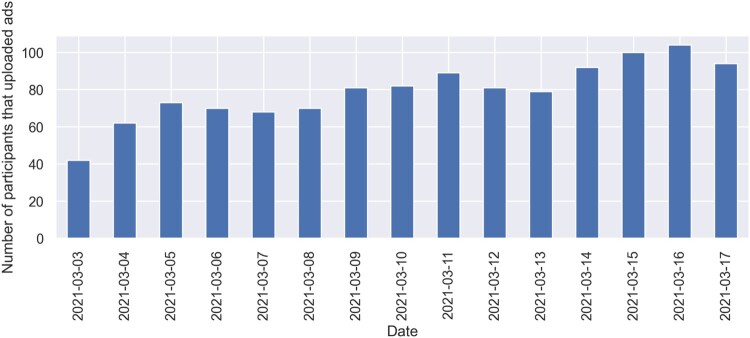
Number of participants who uploaded ads over time.

**Figure I2.**
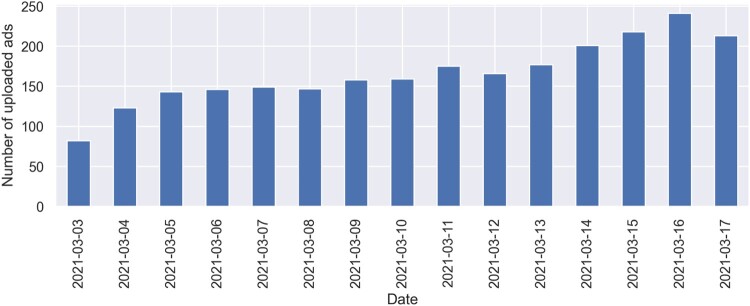
Number of uploaded ads over time.

## Appendix

### Appendix A: Measures

**Table A1.** Measures of issues, dependent variables, moderators, and control variables.

Variable	Items	M	SD
Issue	What do you think are the main problems in our country? (Open question)	0.22	0.41
*Momentary responses*			
Ad perceptions	I find the political ad I just uploaded … (1 = not at all, 7 = very much):		
	- interesting	3.10	1.69
	- informative	3.09	1.69
	- persuasive	2.95	1.66
Party evaluations	I find the party in the political ad … (1 = not at all, 7 = very much):		
	- honest	3.76	1.62
	- competent	3.71	1.63
	- likable	3.27	1.61
*Behavioral outcome*			
Vote choice	Which party did you vote? (0 = did not vote, 1 = voted)	0.13	0.34
*Moderator*			
Party identification	How likely it is that you will vote for . .. (party name)? (0 = will never vote, 11 = will definitely vote)	3.96	3.62
*Control variables*			
Age	What is your age? (Fill in a number)	48.53	16.17
Gender	What is your gender? (0 = Female, 1 = Male)	0.53	0.50
Education	What is your educational level? (1 = Low, 2 = middle, 3 = high)	2.61	0.59

### Appendix B: Summary of the issues in the data

**Table B1.** Number of issues appeared in Facebook political ads and mentioned by participants.

Issue	Description and examples	Appeared in ads	Mentioned by participant
Corona	COVID-19 related issues	140	96
Health	Health and health-related social security	99	71
Environment	Sustainability, climate and energy issues	218	92
Education	Education and student-related issues	96	42
Crime	Law enforcement and criminal justice	34	29
Defense	Military and terrorism	2	4
Economy	Economic situation	33	63
Living	Living environment and rising price of living	4	2
Housing	Housing and housing shortage	80	72
Job	Job market, unemployment and income	88	25
Tax	Tax	30	10
Pension	Pension	56	3
Immigration	Immigration and asylum policy, Islam	57	35
Inequality	Social, income, race and ethnic etc.	66	52
Government	Government debt, corruption, bureaucracy, etc.	28	37
Foreign policy	Foreign policy	2	3
EU*	EU related issues	39	3
Agriculture*	Agriculture	27	4
Animal*	Animal welfare and protection	17	0
Infrastructure*	Public transport, tourism	11	3
Social benefit*	Social benefit and allowance	22	12
Social behavior*	Social order, human behaviors	22	19
Media*	Disinformation, trust in journalism	3	4
Population*	Overpopulation, aging population	0	7

Note*.* *Indicates issues not included in the original codebook but added to the list of issues after coding.

### Appendix C: Number of received online political ads per Dutch party

**Table C1.** Number of received online political ads per Dutch party.

Party name	Number of ads	Number of ads with specific topics	Percentage (%)
CDA	331	190	57.4
VVD	202	28	13.9
D66	202	46	22.8
SP	198	121	61.1
PvdA	193	121	62.7
Volt	165	47	28.5
GroenLinks	155	96	61.9
Forum voor Democratie	125	93	74.4
Partij voor de Dieren	123	93	75.6
JA21	94	63	67.0
ChristenUnie	56	11	19.4
Libertaire Partij	44	32	72.7
BIJ1	40	30	75.0
50 Plus	24	17	70.8
Code Oranje	17	4	23.5
BoerBurgerBeweging	17	14	82.4
DENK	16	6	37.5
Piratenpartij	15	2	13.3
JONG	10	10	100
PVV	10	0	0
SGP	7	1	14.3
Splinter	5	1	20.0
NLBeter	3	1	33.3
Voor 14	2	2	100
NIDA	2	0	0
Oprecht	1	1	100
De Groenen	1	1	100

### Appendix D: Description of Dutch political parties

**Table D1.** Description of Dutch political parties.

Party name	English name	Main ideology
CDA	Christian Democratic Appeal	Christian democracy and social conservatism
VVD	People’s Party for Freedom and Democracy	Conservative liberalism
D66	Democrats 66	Social liberalism and progressivism
SP	Socialist Party	Democratic socialism and left-wing populism
PvdA	Labor Party	Social democracy
Volt	Volt Netherlands	Social liberalism and pro-Europeanism
GroenLinks	Green Left	Green politics and social democracy
Forum voor Democratie	Forum for Democracy	National conservatism and Right-wing populism
Partij voor de Dieren	Party for the Animals	Animal welfare and environmentalism
JA21	Correct Answer 2021	Right-wing populism and anti-immigration
ChristenUnie	Christian Union	Christian democracy and social conservatism
Libertaire Partij	Libertarian Party	Right-libertarianism and economic liberalism
BIJ1	Together	Egalitarianism and anti-racism
50 Plus	50 Plus	Pensioners’ interests and populism
Code Oranje	Code Orange	Direct democracy
BoerBurgerBeweging	Farmer–Citizen Movement	Agrarianism and food politics
DENK	Think	Social democracy and pro-immigration
Piratenpartij	Pirate Party	Open government and freedom of information
JONG	Young	Youth politics
PVV	Party for Freedom	Nationalism, right-wing populism and anti-immigration
SGP	Reformed Political Party	Christian right and social conservatism
Splinter	Splinter	Social liberalism and green politics
NLBeter	Netherlands Better	Public sector interests
Voor 14	For 14	Minimum wage
NIDA	Appeal	Islamic democracy
Oprecht	Sincere	Anti-immigration
De Groenen	The Greens	Green politics

### Appendix F: Overview of variables

**Table F1.** Overview of variables.

Variables	*M*	*SD*	*ICC*	Level	Measurement point	Pearson’s Correlations				
						1	2	3	4	5
1. Congruency	0.22	0.41	.07	Ad	Surveys (1st-4th wave)	-				
2. Ad perceptions	3.05	1.60	.39	Ad	mESM	.06**	-			
3. Party evaluations	3.58	1.53	.34	Ad	mESM	-.02	.73***	-		
4. Vote choice	0.13	0.34	.13	Ad	Survey (3rd wave)	-.01	.30***	.39***	-	
5. Party identification	3.96	3.62	.24	Ad	Survey (4th wave)	-.00	.48***	.67***	.56*** -	-

^∗^ *p < *.1; ^∗∗^*p < *.01; ^∗∗∗^*p < *.001.

### Appendix G: Lavaan syntax of the models

1. Multilevel mediation model:

model1<-’

level: 1

ad_perception ∼ a*congruency_1

party_evaluation ∼ c*congruency_1

w4_vote_choice ∼ e*congruency_1 + b*ad_perception + d*party_evaluation

ad_perception ∼∼ party_evaluation

level: 2

ad_perception ∼ f*congruency_1 + age + gender + education

party_evaluation ∼ h*congruency_1 + age + gender + education

w4_vote_choice ∼ j*congruency_1 + g*ad_perception + i*party_evaluation + age + gender + education

ad_perception ∼∼ party_evaluation

congruency_1 ∼∼ congruency_1

ad_perception ∼∼ ad_perception

party_evaluation ∼∼ party_evaluation

w4_vote_choice ∼∼ w4_vote_choice

# indirect and total effects within-individual

ab:= a * b

cd:= c * d

totalwith:= ab + cd + e

totalwith1:= ab + e

totalwith2:= cd + e

# indirect and total effects between-individual

fg:= f * g

hi:= h * i

totalbw:= fg + hi + j

totalbw1:= fg + j

totalbw2:= hi + j

’

fit1<-sem(model1,data = analysis_df, cluster = ‘user_id’)

summary(fit1,fit.measures = TRUE,standardized = TRUE,rsquare = TRUE)

parameterestimates(fit1, boot.ci.type = ‘bca.simple’, standardized = TRUE) %>%

kable()

2. Multilevel moderated mediation model:

model2<-’

level: 1

ad_perception ∼ a*congruency_1 + w1*ptv + w2*congruency_1:ptv

party_evaluation ∼ c*congruency_1 + x1*ptv + x2*congruency_1:ptv

w4_vote_choice ∼ e*congruency_1 + b*ad_perception + d*party_evaluation

ad_perception ∼∼ party_evaluation

level: 2

ad_perception ∼ f*congruency_1 + y1*ptv + y2*congruency_1:ptv + age + gender + education

party_evaluation ∼ h*congruency_1 + z1*ptv + z2*congruency_1:ptv + age + gender + education

w4_vote_choice ∼ j*congruency_1 + g*ad_perception + i*party_evaluation + age + gender + education

ad_perception ∼∼ party_evaluation

congruency_1 ∼∼ congruency_1

ad_perception ∼∼ ad_perception

party_evaluation ∼∼ party_evaluation

w4_vote_choice ∼∼ w4_vote_choice

# indirect and total effects within-individual

ab:= a * b

cd:= c * d

totalwith:= ab + cd + e

totalwith1:= ab + e

totalwith2:= cd + e

# indirect and total effects between-individual

fg:= f * g

hi:= h * i

totalbw:= fg + hi + j

totalbw1:= fg + j

totalbw2:= hi + j

’

fit2<-sem(model2,data = analysis_df, cluster = ‘user_id’)

summary(fit2,fit.measures = TRUE,standardized = TRUE,rsquare = TRUE)

parameterestimates(fit2, boot.ci.type = ‘bca.simple’, standardized = TRUE) %>%

kable()

### Appendix H: Robustness check

As a robustness check, five independent multilevel regression analyses were conducted using STATA. These five analyses tested the direct effects of issue congruency and the moderating effect of party identification. We observed findings identical to what we found with multilevel (moderated) mediation models. Table H1 shows the results of the (moderated) direct effects. Specifically, the main effect of issue congruency on vote choice was not supported (*b *= 0.001, *p *= 0.953). However, we observed a positive effect of issue congruency on ad perception (*b *= 0.252, *p *< .001), while the interacting effect of party identification was not found (*b *= 0.028, *p *= .099). Moreover, issue congruency had no significant impact on party evaluation (*b *= −0.048, *p *= .511), but party identification played a moderating role in this relationship (*b *= 0.053, *p *< .001), meaning that a congruent ad leads to a more positive party evaluation only when the ad promotes a more favorable party. The indirect effects of issue congruency on vote choice via ad perception and party evaluation were also tested. The resutls showed that vote choice can be positively affected by ad perception (*b *= 0.063, *p *< .001) and party evaluation (*b *= 0.086, *p *< .001).

**Table H1.** Multilevel regressions predicting momentary responses and voting behavior.

	Ad perception		Party evaluation		Vote choice
	Direct effect	Moderating effect	Direct effect	Moderating effect	Direct effect
	B(SE)	B(SE)	B(SE)	B(SE)	B(SE)
Constant	2.550 (0.362)***	1.568 (0.332)***	3.720 (0.326)***	2.403 (0.276)***	0.110 (0.053)*
Issue congruency	0.252 (0.071)***	0.136 (0.091)	−0.048 (0.072)	−0.263 (0.077) ***	0.001 (0.018)
Party identification		0.196 (0.009)***		0.270 (0.007) ***	
IC * PID		0.028 (0.169)		0.053 (0.014)***	
Age	0.004 (0.007)	0.009 (0.006)	−0.005 (0.006)	0.002 (0.005)	0.000 (0.001)
Gender	0.143 (0.207)	0.081 (0.189)	0.321 (0.187)	0.245 (0.157)	0.044 (0.031)
Education	0.129 (0.118)	0.115 (0.108)	0.014 (0.107)	−0.026 (0.089)	0.000 (0.017)
N of observations	2034	1936	2033	1935	2035
N of respondents	140	138	140	138	140
Log likelihood	−3479.867	−2999.983	−3492.121	−2668.561	−622.543

Note*.* IC = issue congruency, PID = party identification. ^∗^*p < *.1; ^∗∗^*p < *.01; ^∗∗∗^*p < *.001.

### Appendix I: Information regarding the study participation

Participants received ads from diverse parties: 16.4 percent received political ads from only one party, 37.9 percent received ads from two to five parties, and 45.7 percent received ads from more than five parties. Considering only some big parties disseminated ads in the hot phase of the election campaign (see *Appendix C*), this finding indicated that participants were not specifically targeted by political parties.

Besides, we measured participants’ motivation to participate in the study (1 = ‘not motivated at all’, 7 = ‘very motivated’) in two short surveys respectively at the start and end of the mESM study. The results showed that participants were slightly more motivated by the end of the study (*b* = 0.505, *p *= .018). We also asked participants to estimate how many political ads they had seen but not sent during the mESM study. 54.1 percent reported that they uploaded all the ads they encountered every day, 38.5 percent reported that the number of ads they had seen without uploading was less than five, 4.9 percent reported six to ten, and 2.5 percent reported more than ten. Interestingly, we found that as it got closer to election day, more participants started uploading ads, and more ads were uploaded (see Figure I1 and Figure I2), which is in line with the findings of previous studies that the number of political ads ramped up toward the end of the election campaign in order to boost the turnout (Ridout et al., 2021). A positive correlation between participants’ Facebook use and the number of ads they uploaded was also found (r = . 21, *p* = . 013).
